# The correlations between DNA methylation and polymorphisms in the promoter region of the human telomerase reverse transcriptase (hTERT) gene with postoperative recurrence in patients with thyroid carcinoma (TC)

**DOI:** 10.1186/s12957-017-1170-z

**Published:** 2017-06-06

**Authors:** Jian-Jun Li, Ping Chen Jue-Ru Zheng, Yao-Zong Wang

**Affiliations:** Thyroid Surgery, Ningbo No.2 Hospital, No. 41, Xibei Street, Haishu District, Ningbo, 315010 Zhejiang People’s Republic of China

**Keywords:** Human telomerase reverse transcriptase gene, Thyroid carcinoma, DNA methylation, Polymorphism, Postoperative recurrence

## Abstract

**Background:**

This study aims at exploring the correlations between DNA methylation and polymorphisms in the promoter region of the human telomerase reverse transcriptase (hTERT) gene and postoperative recurrence in patients with thyroid carcinoma (TC).

**Methods:**

A total of 312 patients diagnosed with TC were chosen for the study and categorized into recurrence (*n* = 75) and non-recurrence (*n* = 237) groups. The hTERT rs2736100 and rs2736098 polymorphisms were detected by performing polymerase chain reaction-restriction fragment length polymorphism. DNA methylation in the promoter region of hTERT gene was evaluated by pyrosequencing. A telephonic and/or outpatient follow-up was conducted for all patients. The correlations of DNA methylation and polymorphisms in the promoter region of hTERT with postoperative recurrence of TC patients underwent analysis.

**Results:**

The patient in the recurrence group showed evidently different pathological types and tumor stages in comparison to the non-recurrence group. The GG genotype of hTERT rs2736100 might increase the recurrence risk of TC patients. No correlations between hTERT rs2736098 polymorphisms and recurrence risk were observed. Compared to the TT + TG genotype frequency, the rs2736100 GG genotype frequency increased in patients without multicentricity, patients with extrathyroidal invasion, patients with lymph node metastasis, patients with undifferentiated carcinoma, and patients in the III + IV stage. The recurrence group showed significantly higher DNA methylation level compared to the non-recurrence group. The DNA methylation level was closely associated to tumor stage and lymph node metastasis of TC patients in the recurrence group.

**Conclusions:**

The DNA methylation and rs2736100 polymorphisms in the promoter region of hTERT gene might be in correlation to postoperative recurrence of TC patients.

## Background

Thyroid carcinoma (TC) is an uncommon form of carcinoma but is one of the most common malignancies in the human endocrine system [1]. It accounts for about 2% of newly diagnosed carcinoma cases and a majority of deaths related to human endocrine carcinoma every year [2]. TC still remains as the most common type of cancer that is primarily diagnosed in adolescence and young female adults aged between 15~29 years and second between 30~39 years [3]. In recent years, with the rapidly expanding prevalence, the treatment of TC has been one of the most researched subjects in the Department of Head and Neck Surgery [4]. A neck dissection is usually a safe therapeutic treatment for TC patients but can induce some complications [5]. The 5- and 10-year survival rates of most TC patients are excellent, but it is another type of complication in patients with poorly differentiated TC [6]. Therefore, the development of novel prognostic biomarkers is essential for TC [1]. On the other hand, the increasing evidence suggested that the common variant in the DNA sequence, especially single-nucleotide polymorphisms (SNPs), is commonly associated to the efficacy of surgical treatment [7].

Telomerase is an enzyme, which accounts for the maintenance of chromosomal end integrity, and plays a crucial role in tumorigenesis and progression of cancers; its activity is characterized by the expression of the human telomerase reverse transcriptase (hTERT) gene [8]. The hTERT gene being the key component of telomerase catalytic activity, encodes the reverse transcriptase component of the telomerase complex, and its abnormity is considered to be associated to tumorigenesis [9, 10]. High hTERT expression and telomerase activity have been found in thyroid tumors particularly in advanced forms, while in the majority of normal adult tissues, including normal thyroid tissues, telomerase activity is close to none [11]. DNA methylation is generally reported to be closely related to gene repression through the prevention of activating transcription factors against binding to the DNA [12]. The promoter of the hTERT gene encodes the catalytic subunit of the telomerase enzyme and has an intensive CG-rich cytidine-phosphate-guanosine (CpG) island, suggesting the crucial role of methylation in the regulation of hTERT expression [13]. The rs2736100 polymorphism is located on intron 2 of the hTERT gene, and the rs2736098 polymorphism is localized on exon 2 of hTERT gene [14, 15]. Recently, despite the inconclusive results, relations between these SNPs and cancer risk have been reported [16]. It has been established that the hTERT promoter mutations are linked to poor prognosis of several human tumors, which include gliomas, ovarian cancer, and melanoma [17–20]. Further, the mutations in the promoter region of hTERT are extremely prevalent in advanced TC, and an acquisition of such a mutation could activate the accumulation of excess genetic defects, which result in disease progression [11]. Therefore, in this study, we aimed at examining the correlations between DNA methylation and polymorphisms in the promoter region of the hTERT gene and postoperative recurrence in patients with TC.

## Methods

### Study subjects

From April 2007 to March 2012, a total of 312 patients who were histologically and radiologically diagnosed with TC and treated in the Ningbo No.2 Hospital (*n* = 165) and Ningbo No.7 Hospital (*n* = 147) were selected for this retrospective study. The diagnosis was conducted in compliance with the guidelines published by the American Thyroid Association (ATA) in 2015 [21]. All subjects were in strict accordance with the inclusion and exclusion criteria [22–24]. The inclusion criteria were as follows: (1) patients recently diagnosed with TC, (2) patients ≥18 years old, (3) patients with definitive pathological diagnosis, and (4) patients with no history of other tumors. Exclusion criteria were as follows: (1) patients with incomplete pathological data and (2) patients suffering from severe liver or kidney disease, cardiovascular disease, hematologic disease, or malignant tumor before admission. After treatment, the patients were allotted into the recurrence group (patients who showed postoperative recurrence, *n* = 75) or the non-recurrence group (patients who showed no postoperative recurrence, *n* = 237). The study was performed with the approval from the Ethics Committee of the Ningbo No.2 Hospital. All patients signed informal consent.

### Treatment for TC

TC on the basis of their behavior and histology can be classified into differentiated and undifferentiated cancers. The surgical options of treatment for differentiated TC include total/near-total thyroidectomy, subtotal thyroidectomy, as well as lobectomy plus isthmusectomy [25]. In our study, there were 28 TC patients with a tumor of diameter 3 cm and they were treated by total/near-total thyroidectomy or subtotal thyroidectomy; among them, 7 cases by lymph node metastasis and 3 cases with pulmonary metastasis were treated by thyroidectomy. For the three cases with a tumor of diameter 4 cm, whose thyroid capsule was invaded by tumor cells were treated by total thyroidectomy plus bilateral lymph node dissection and mediastinal lymph node dissection. The five cases with thyroid microcarcinoma (tumor diameter <1 cm) were treated by unilateral thyroidectomy. The subtotal thyroidectomy plus bilateral lymph node dissection and mediastinal lymph node dissection was the treatment for TC patients with a tumor diameter between 1 and 3 cm. For undifferentiated TC, surgery still remains as the main treatment during the early stage and radiotherapy and chemotherapy during the end stage. All patients acquired some routine nursing care in the Department of General Surgery and strengthening of perioperative nursing intervention.

### Polymerase chain reaction-restriction fragment length polymorphism (PCR-RFLP)

Peripheral venous blood samples were withdrawn from all patients. The genomic DNA was then extracted from the blood samples with a Qiagen DNA extraction kit (Qiagen, Valencia, CA, USA). Two loci (rs2736100 and rs2736098) were chosen in the promoter region of the hTERT gene. The primers of the two loci for polymerase chain reaction (PCR) are shown in Table 1. The PCR conditions for these two loci were different. PCR condition for rs2736100 were as follwos: pre-denaturation at 94 °C for 5 min; 35 cycles of denaturation at 94 °C for 30 s, annealing at 63 °C for 30 s, and extension at 72 °C for 45 s; and extension at 72 °C for 10 min. PCR condition for rs2736098 were as follows: pre-denaturation at 94 °C for 5 min; 35 cycles of denaturation at 94 °C for 30 s, annealing at 61 °C for 30 s, and extension at 72 °C for 45 s; and extension at 72 °C for 10 min. Their PCR products were preserved at 4 °C. Identification of genotypes was done by enzyme digestion. The hTERT gene polymorphism in the promoter region was detected by PCR-RFLP.

**Table 1:** Primer sequences of rs2736100 and rs2736098 polymorphisms for polymerase chain reaction

SNP	Primer sequence
rs2736100	Forward: 5′-CCCCACAAGCTAAGCATTAT-3′
	Reverse: 5′-GAAGAACCACGCAAAGGAC-3′
rs2736098	Forward: 5′-CCGTGGTTTCTGTGTGGTGT-3′
	Reverse: 5′-CCCCTGAGGAGTAGAGGAAGT-3′

*SNP* single-nucleotide polymorphism

### Pyrosequencing

The PyroMark Q24 pyrosequencing instrument, EpiTect Bisulfite Kit, and Hostart Taq PCR mix (all acquired from Qiagen, Valencia, CA, USA) were all applied for determining the DNA methylation level in the promoter region of the hTERT gene. The sequences were amplified after the transformation and purification of the DNA samples with sulfite. Then the amplified DNA was detected by pyrosequencing with the primers mentioned in Table 2.

**Table 2:** Primer sequences for polymerase chain reaction

Primer	Primer sequence
TERTMF	5′-ATGATGTGGAGGTTTTGGGAATAG-3′
TERTMR	5′-Biotin-CCCAACCTAAAAACAACCCTAAT-3′
TERTS	5′-GGAGGTTTTGGGAATAG-3′

### Follow-up

A telephonic and/or outpatient follow-up for all patients (*n* = 165) was conducted after operation and radiation therapy. The postoperative recurrence was observed and recorded. The postoperative recurrence was identified on the basis of (1) if the hormone levels in the blood were higher than the normal values and (2) observation by radiological re-examination.

### Statistical analysis

All data was processed with the SPSS 20.0 software (SPSS Inc.; Chicago, IL, USA). The measured data was expressed as mean value ± standard deviation (mean ± SD) and was analyzed by the *t* test. The enumeration data was expressed in percentage and was analyzed by the chi-squared test. Hardy-Weinberg equilibrium was used for analyzing the frequency distribution of the genotypes of the hTERT gene in the recurrence and non-recurrence groups. The recurrence risk was evaluated with odds ratio (OR) and 95% confidence interval (95%CI). *P* < 0.05 was considered to be statistically significant. Finally, for evaluation of the sample size, G*power software was used for power calculation, sample size and *P* values were used for estimating the statistical test of effectiveness, and effect size (ES) was employed for comparing the differences [26, 27].

## Results

### Baseline characteristics of TC patients in the recurrence and non-recurrence groups

The baseline characteristics of TC patients were compared in between the recurrence and non-recurrence groups (Table 3). The recurrence group (*n* = 75) included 24 male and 51 female patients with the mean age as 45.04 ± 10.53 years. The non-recurrence group (*n* = 237) included 67 male and 170 female patients with the mean age as 42.58 ± 8.40 years. No significant difference in the parameters of gender and mean age were observed in between these two groups (both *P* > 0.05). The pathological type and tumor stage of the recurrence group were evidently different in comparison to the non-recurrence group (both *P* < 0.05), which indicated that the postoperative recurrence of TC might be associated to the pathological type and tumor stage.

**Table 3:** Baseline characteristics of patients between the recurrence and non-recurrence groups

Characteristic	Non-recurrence group	Recurrence group	*χ* ^2^/*t*	*P*
	(*n* = 237)	(*n* = 75)		
Gender				
Male	67 (28.3)	24 (32.0)	0.69	0.467
Female	170 (71.7)	51 (68.0)		
Mean age (years)	42.58 ± 8.40	45.04 ± 10.53	1.26	0.211
Pathological type				
Papillary thyroid carcinoma	220 (92.8)	41 (54.7)	72.13	<0.0001
Follicular thyroid carcinoma	10 (4.2)	7 (9.3)		
Medullary thyroid carcinoma	5 (2.1)	10 (13.3)		
Undifferentiated carcinoma	2 (0.8)	17 (22.7)		
Tumor stage				
I	174 (73.4)	10 (13.3)	98.67	< 0.0001
II	25 (10.5)	20 (26.7)		
III	35 (14.8)	29 (38.7)		
IV	3 (1.3)	16 (21.3)		

### Genotype determination of hTERT rs2736100 and rs2736098

**Fig. 1:**
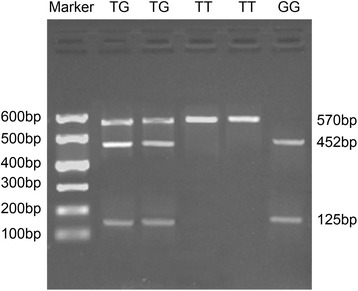
Genotypes of hTERT rs2736100 identified by enzyme digestion. Note: *hTERT* human telomerase reverse transcriptase

**Fig. 2:**
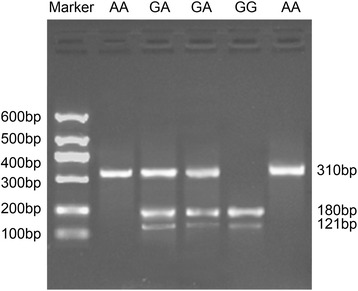
Genotypes of hTERT rs2736098 identified by enzyme digestion. Note: *hTERT* human telomerase reverse transcriptase

The genotyping of hTERT rs2736100 and rs2736098 was identified using enzyme digestion. For rs2736100 (T > G), there were three bands for the TG genotype (125, 452, and 570 bp), one band for the TT genotype (570 bp) and two bands for the GG genotype (125 and 452 bp), as shown in Fig. 1. For rs2736098 (G > A), there were two bands for the GG genotype (121,and 180 bp), one band for the AA genotype (310 bp) and three bands for the GA genotype (121, 180 and 310 bp), as shown in Fig. 2.

### Allele frequency and genotype distribution of hTERT rs2736100 and rs2736098 between the recurrence and non-recurrence groups

The allele and genotype distribution of hTERT rs2736100 and rs2736098 corresponded to the Hardy-Weinberg equilibrium (*P* > 0.05). Therefore, the samples in our study were random and representative.

The hTERT rs2736100 frequency of T allele was 50.8% in the non-recurrence group and 35.3% in the recurrence group, and the frequency of G allele was 49.2 vs 64.7%; which differed significantly between the recurrence group and the non-recurrence group (*P* = 0.001). In the non-recurrence group, the TT, TG, and GG genotype frequencies of hTERT rs2736100 were 22.8, 56.1, and 21.1%, respectively. In the recurrence group, the TT, TG, and GG genotype frequencies were 9.3, 52.0, and 38.7%, respectively. The frequency of hTERT rs2736098 G allele was 75.1 vs. 78.7% in between the non-recurrence and recurrence groups, and of A allele was 24.9 vs. 21.3%. In the non-recurrence group, the GG, GA, and AA genotype frequencies of hTERT rs2736098 were 57.0, 36.3, and 6.8%, respectively. In the recurrence group, the GG, GA, and AA genotype frequencies were 61.3, 34.7, and 4.0% respectively. No significant differences observed in the non-recurrence and recurrence groups (all *P* > 0.05). The GG genotype of hTERT rs2736100 can potentially increase the recurring risk of TC (OR = 4.47, 95%CI = 1.80 ~ 11.12). There was no relation between hTERT rs2736098 and recurrence of TC (Table 4).

**Table 4:** Allele frequency and genotype distribution of hTERT rs2736100 and rs2736098 polymorphisms between the recurrence and non-recurrence groups

	Non-recurrence group	Recurrence group	*P*	OR (95%CI)
	(*n* = 237)	(*n* = 75)		
rs2736100				
TT genotype	54 (22.8)	7 (9.3)	Ref	
TG genotype	133 (56.1)	39 (52.0)	0.059	0.44 (0.19–1.05)
GG genotype	50 (21.1)	29 (38.7)	0.001	4.47 (1.80–11.12)
T allele	241 (50.8)	53 (35.3)	Ref	
G allele	233 (49.2)	97 (64.7)	0.001	1.89 (1.29–2.77)
rs2736098				
GG genotype	135 (57.0)	46 (61.3)	Ref	
GA genotype	86 (36.3)	26 (34.7)	0.671	0.89 (0.51–1.54)
AA genotype	16 (6.8)	3 (4.0)	0.353	0.55 (0.15–1.98)
G allele	356 (75.1)	118 (78.7)	Ref	
A allele	118 (24.9)	32 (21.3)	0.373	0.82 (0.53–1.27)

*hTERT* human telomerase reverse transcriptase, *Ref* reference, *OR* odds ratio, *CI* confidence interval

### Correlations of hTERT rs2736100 polymorphism with baseline characteristics of TC patients in the recurrence and non-recurrence groups

As shown in Table 5, hTERT rs2736100 polymorphism was correlated to multicentricity, extrathyroidal invasion, lymph node metastasis, and pathological type of patients in the recurrence group (all *P* < 0.05), while no correlation was observed in between hTERT rs2736100 polymorphism and baseline characteristics in the non-recurrence group patients. The frequency of rs2736100 GG genotype increased in patients without multicentricity, patients with extrathyroidal invasion, patients with lymph node metastasis, and patients with undifferentiated carcinoma, in comparison to the patients with multicentricity, patients without extrathyroidal invasion, patients without lymph node metastasis, or patients with differentiated carcinoma (69.0, 75.9, 79.3, and 51.7 vs 31.0, 24.1, 48.3, and 0.0%) (*P* = 0.020, *P* < 0.001, *P* < 0.028, and *P* < 0.004).

**Table 5:** Correlations of hTERT rs2736100 polymorphism with baseline characteristics of patients in the recurrence and non-recurrence groups

Characteristic	Non-recurrence group			Recurrence group		
	TT + TG (%)	GG (%)	*P*	TT + TG (%)	GG (%)	*P*
	187	50		46	29	
Tumor size						
<20 mm	127 (67.9)	30 (60.0)	0.293	31 (67.4)	16 (55.2)	0.287
≥20 mm	60 (32.1)	20 (40.0)		15 (32.6)	13 (44.8)	
Multicentricity						
Yes	115 (61.5)	28 (56.0)	0.532	27 (58.7)	9 (31.0)	0.02
No	72 (38.5)	14 (28.0)		19 (41.3)	20 (69.0)	
Extrathyroidal invasion						
Yes	66 (35.3)	22 (44.0)	0.258	11 (23.9)	22 (75.9)	<0.001
No	121 (64.7)	28 (56.0)		35 (76.1)	7 (24.1)	
Lymph node metastasis						
Yes	33 (17.6)	11 (22.0)	0.482	25 (54.3)	23 (79.3)	0.028
No	154 (82.4)	39 (78.0)		21 (45.7)	6 (20.7)	
Pathological type						
Papillary thyroid carcinoma	173 (92.5)	47 (94.0)	0.907	27 (58.7)	10 (34.5)	0.004
Follicular thyroid carcinoma	8 (4.3)	2 (4.0)		5 (10.9)	2 (6.9)	
Medullary thyroid carcinoma	4 (2.1)	1 (2.0)		8 (17.4)	2 (6.9)	
Undifferentiated carcinoma	2 (1.1)	0		6 (13.0)	15 (51.7)	
Tumor stage						
I + II	156 (83.4)	43 (86.0)	0.659	21 (45.7)	9 (31.0)	
III + IV	31 (16.6)	7 (14.0)		25 (54.3)	20 (69.0)	0.208

*hTERT* human telomerase reverse transcriptase

### DNA methylation level in the promoter region of hTERT gene in the recurrence and non-recurrence groups

**Fig. 3:**
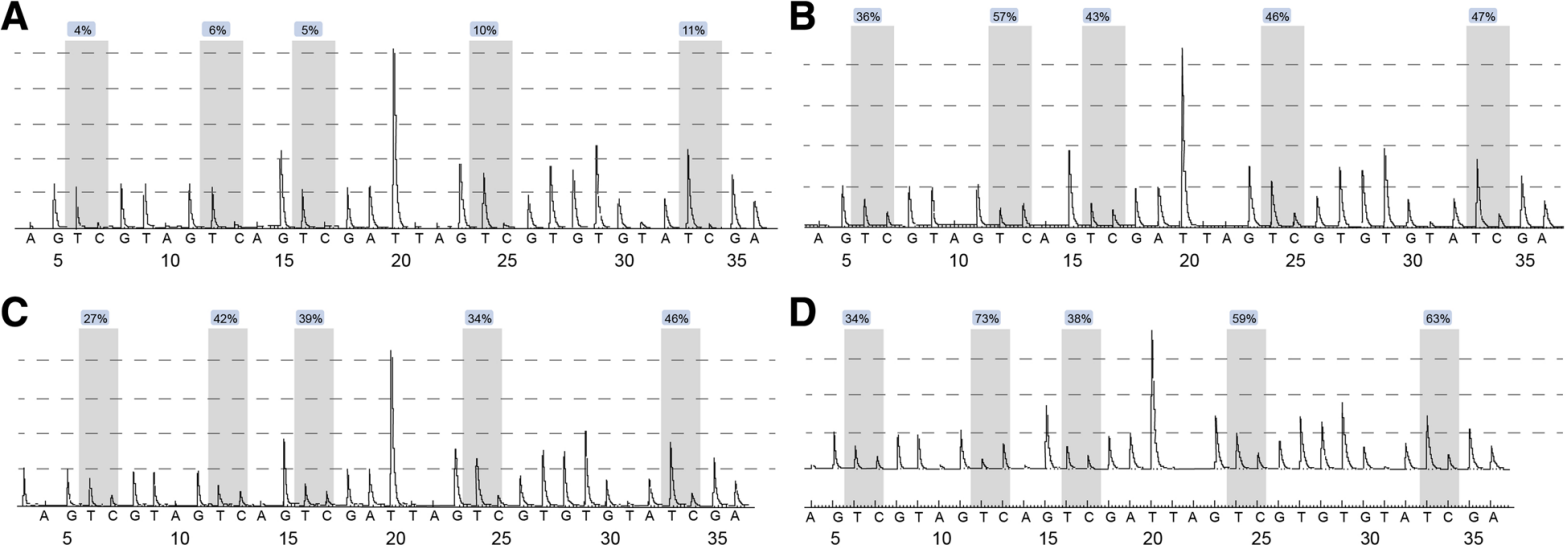
**a**–**d** DNA methylation levels in the promoter region of the hTERT gene of the four TC samples evaluated by pyrosequencing. Note: *hTERT*, human telomerase reverse transcriptase; *TC*, thyroid carcinoma

Five cytidine-phosphate-guanosine (CpG) sites were identified by pyrosequencing of the promoter region of hTERT upstream of the transcription start site (UTSS) region. Figure 3a–d as the pyrosequencing diagrams of the four TC samples, respectively. The DNA methylation levels of these five CpG sites are shown in Fig. 3. The percentage in the blue frame presented the DNA methylation level of the corresponding site. Different DNA methylation levels were observed in different samples.

**Fig. 4:**
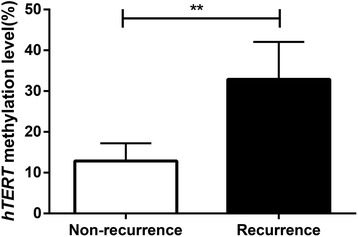
DNA methylation levels in the hTERT UTSS promoter region of patients of the recurrence and non-recurrence groups. Note: *hTERT* human telomerase reverse transcriptase, *UTSS* upstream of the transcription start site; **P < 0.05 compared to the non-recurrence group

The DNA methylation levels of five CpG sites in the hTERT UTSS promoter region were evaluated by pyrosequencing of all 312 patients. A percentage higher than 5% is considered as an indication of DNA methylation [28]. The DNA methylation levels in the hTERT UTSS promoter region were observed to be higher than 5% in all 312 samples. The DNA methylation levels and the mean DNA methylation levels at the five CpG sites were significantly higher in the recurrence group in comparison to the non-recurrence group (Fig. 4 and Table 6).

**Table 6:** Comparison of methylation levels at five CpG sites of patients in the recurrence and non-recurrence groups

Group	CpG 1	CpG 2	CpG 3	CpG 4	CpG 5	Methylation levels	Mean value	*P*
Non-recurrence group	16%	17%	18%	9%	10%	Low	14.0%	<0.001
Recurrence group	25%	31%	21%	39%	38%	High	30.8%	

*CpG* cytidine-phosphate-guanosine

### Correlations of DNA methylation level in the promoter region of hTERT gene with baseline characteristics of TC patients in the recurrence and non-recurrence groups

The quartile (17%) of mean DNA methylation levels was considered as a threshold of hypomethylation and hypermethylation [28]. As shown in Table 7, the DNA methylation level of rs2736100 is closely associated to tumor stage and lymph node metastasis of TC patients in the recurrence group (both *P* < 0.05) and not correlated to the non-recurrence group patients (both *P* > 0.05). Increased hypermethylation of rs2736100 was observed in TC patients with lymph node metastasis and patients in the III + IV stage in comparison to the patients without lymph node metastasis and those in the I + II stage in the recurrence group (77.4 and 69.8 vs. 22.6 and 30.2%) (*P* < 0.001, *P* = 0.007).

**Table 7:** Correlations of methylation levels of the promoter region of hTERT gene with baseline characteristics of patients in the recurrence and non-recurrence groups

Characteristic	Non-recurrence group			Recurrence group		
	Hypomethylation (%)	Hypermethylation (%)	*P*	Hypomethylation (%) (*n* = 22)	Hypomethylation (%) (*n* = 53)	*P*
Tumor size						
<20 mm	112 (70.0)	45 (58.4)	0.078	12 (54.5)	19 (35.8)	
≥20 mm	48 (30.0)	32 (41.6)		10 (45.5)	34 (64.2)	0.134
Multicentricity						
Yes	102 (63.8)	41 (53.2)	0.122	9 (40.9)	27 (50.9)	
No	58 (36.3)	36 (46.8)		13 (59.1)	26 (49.1)	0.428
Extrathyroidal invasion						
Yes	57 (35.6)	31 (40.3)	0.489	8 (36.4)	25 (47.2)	0.391
No	103 (64.4)	46 (59.7)		14 (63.6)	28 (52.8)	
Lymph node metastasis						
Yes	29 (18.1)	15 (19.5)	0.802	7 (31.8)	41 (77.4)	<0.001
No	131 (81.9)	62 (80.5)		15 (68.2)	12 (22.6)	
Pathological type						
Papillary thyroid carcinoma	147 (91.9)	73 (94.8)	0.707	12 (54.5)	25 (47.2)	
Follicular thyroid carcinoma	7 (4.4)	3 (3.9)		2 (9.1)	5 (9.4)	
Medullary thyroid carcinoma	4 (2.5)	1 (1.3)		2 (9.1)	8 (15.1)	
Undifferentiated carcinoma	2 (1.3)	0 (0.0)		6 (27.3)	15 (28.3)	0.897
Tumor stage						
I + II	135 (84.4)	64 (83.1)	0.805	14 (63.6)	16 (30.2)	
III + IV	25 (15.6)	13 (16.9)		8 (36.4)	37 (69.8)	0.007

*hTERT* human telomerase reverse transcriptase

## Discussion

Neck surgery is thought of as a safe therapeutic method that can bring excellent 5- and 10- year survival rates for TC patients [6]. However, there are still some complications and a relatively high incidence of recurrence after surgery [5]. Therefore, it is of great significance to develop better prognostic biomarkers for treating TC. Our findings have provided evidence that rs2736100 polymorphism and DNA methylation in the promoter region of the hTERT gene is correlated to the prognosis of TC after surgery.

Interestingly, the rs2736100 polymorphism was identified in the promoter region of the hTERT gene and was considerably associated to postoperative recurrence of TC patients. It has been reported that hTERT promoter mutations can increase the transcriptional activity from the promoter [29], which can change the stringency with which hTERT is regulated, which can possibly facilitate the activation of hTERT and consequently lead to a poor outcome of cancers [30]. In line with our study, Xing et al. demonstrated that mutations and gene copy number gain are a representation of some new prognostic markers for TC [31]. Landa et al. found that the hTERT promoter mutations are highly abundant in advanced TC [11]. Additionally, the rs2736100 polymorphism has been reported to be associated to risk of glioma, lung cancer, and testicular germ cell cancer [18, 32, 33]. It has been proven that rs2736100 polymorphism of the hTERT gene has a genotype-specific effect on hTERT expression, which is closely associated to susceptibility to papillary TC [34] Furthermore, our study discovered that the T and G alleles of rs2736100 polymorphism of hTERT gene were associated to the recurrence of TC by PCR-RFLP analysis, and GG genotype can increase the risk of the recurrence of TC.

Moreover, the pyrosequencing assay was performed in our study for analyzing the DNA methylation levels at the five CpG sites, which showed that the DNA methylation levels were significantly higher in the recurrence group compared to the non-recurrence group. An increase in the hypermethylation of rs2736100 was observed in TC patients with lymph node metastasis and patients in the III + IV stage compared to the patients without lymph node metastasis and patients in the I + II stage in the recurrence group. DNA methylation is one of the mechanisms that can instigate gene silencing in the process of carcinogenesis, especially in tumor suppressor genes [35]. Expression of hTERT still remains as one of the major limiting factors for telomerase activity [36]. Expression of hTERT decreased during cell differentiation, and it silenced in completely differentiated somatic cells, while it is frequently was reactivated by means of an unknown mechanism in 80–95% of cancers and immortalized cells, which enabled these cells to be alive with shortened telomeres [37]. Dense hypermethylation was observed in the promoter region of hTERT in most cancer cell lines as reported previously [38–40]. DNA methylation is considered to play a critical role in the regulation of the hTERT gene [8], which can provide us a promising explanation about how the hTERT gene regulated by DNA methylation affects the risk of TC. Aberrant gene methylation has been proposed to be a new prognostic marker for TC [31]. Accumulating studies have provided enough evidence on how DNA methylation and gene polymorphisms are commonly linked to the differences in surgery efficacy for many cancers [41–44].

Our results also revealed that postoperative recurrence of TC was associated to pathologic types and tumor stages and that the DNA methylation levels and the rs2736100 polymorphisms of hTERT gene were associated to the tumor size, multicentricity, extrathyroidal invasion, pathologic types, lymph node metastases, and tumor stage of the recurrence group. Recent studies have demonstrated that tumor size, nodal metastases, and histological type are often associated to postoperative recurrence of TC [30, 45, 46]. In addition to this, Gandolfi et al. revealed that mutations in the hTERT promoter are closely associated to metastasis in papillary TC [47].

## Conclusions

To summarize, our study demonstrated that rs2736100 polymorphism and DNA methylation in the promoter region of the hTERT gene might be correlated to the prognosis of TC after surgery. Our study provided references for the treatment of TC in the aspect of molecular biology. However, it must be noted that our findings need to be further validated by well-designed larger molecular epidemiological researches.

## Abbreviations

CpG: Cytidine-phosphate-guanosine
hTERT: Human telomerase reverse transcriptase
OR: Odds ratio
PCR: Polymerase chain reaction
TC: Thyroid carcinoma
UTSS: Upstream of the transcription start site
